# Omni-channel retail operations in the presence of strategic customers: The benefit of inventory commitment

**DOI:** 10.1371/journal.pone.0264900

**Published:** 2022-05-05

**Authors:** Hongxuan Li, Fan Wu

**Affiliations:** 1 School of Economics and Management, Wuhan University, Wuhan City, Hubei Province, China; 2 School of Economics and Management, Nanchang University, Nanchang City, Jiangxi Province, China; University of Oklahama Norman Campus: The University of Oklahoma, UNITED STATES

## Abstract

This paper studies the impact of strategic customer behavior on retailers’ omni-channel strategies. Customers in the market are classified into strategic omni-channel customers and myopic B&M (brick and mortar) store customers. The retailer firstly charges a full price but will then salvage the leftover inventory at a lower price (markdown policy) for strategic omni-channel customers after the customers’ demand is realized. The strategic omni-channel customers choose purchase timing to maximize their own expected profit. While, the myopic B&M store customers do not choose purchase timing. By characterizing rational expectation equilibrium, we find that the inventory level under markdown policy is higher than that in the classic model where no markdown policy is implemented. Also, the policy will transfer more strategic customers to the online channel to buy at full price so that the retailer will benefit from it. Besides, inventory commitment policy can further increase retailer’s profit based on markdown policy. In addition, we extend the model to the supply chain and present the contrasting view that the total profit of the decentralized supply chain under the wholesale price contract is higher than that of the centralized supply chain. The influence of strategic customer behavior and system parameters on the retailer’s optimal decision is discussed through numerical study.

## 1 Introduction

The popularity of the Internet and intelligent terminals has significantly impacted business, especially on the retail industry. Many retailers have realized that they need to integrate their existing channels to enrich customers’ shopping methods, improve their operational efficiency, and enhance brand competitiveness. Thereby, gradually bringing omni-channel retail into notice. Omni-channel’s purpose is to provide customers with a seamless shopping experience through all available shopping channels [1–4]. For instance, Xiaomi, a company that started with e-commerce, pays more attention to building entity stores called Mi Home where customers can experience and buy new products. There are three main models of omni-channel sales: buy online and pick up in-store (BOPS), buy online and ship to store (BOSS), and buy online ship from a store (BOFS) [5]. Moreover, among all omni-channel fulfillment initiatives, allowing customers to buy online and pick up in-store (BOPS) is regarded as the most important one [6]. Uniqlo, the famous fashion fast-moving brand, is one of the most successful retailers implementing the BOPS strategy. In Tmall’s 2017 and 2018 Double Eleven, Uniqlo’es sales increased by 100 million in 1 minute and 35 seconds relied on more than 500 stores across the country with the BOPS strategy. JDA’s 2016 consumer survey revealed that 46% of respondents had used BOPS options in the past 12 months and that among Best Buy customers, 40% prefered to purchase online and picked up in-store [7].

Because of online shopping and omni-channel strategies, retailers face the pressure of competition, and price wars have always been the most important methods of competition for the market. Just as Tmall will provide discounts and coupons in Double Eleven Shopping Festival, retailers will also organize discount and cashback activities in the shopping festival to attract customer flow. Due to online discount, more and more customers choose to postpone their purchases and buy on sale. Customers are becoming more and more rational, they compare the value that they can obtain with different prices and determine their purchase timing. Retailers certainly recognize the fact that customers often act strategically when purchasing products. Executives at Federated Department Stores, America’s largest operator of department stores, have claimed that the department store industry’s habit of running frequent promotional sales has “trained our customers to only buy on sale”. Such behavior is evident in aggregate data, too; for example, at Wal-Mart, the world’s largest retailer, holiday season sales, including after- Thanksgiving and post-Christmas season sales events, account for close to 20% of total annual sales [8]. Best Buy’s CEO calls those nonstrategic high-end consumers angels who boost profits at the consumer-electronics giant by snapping up high-definition televisions, portable electronics, and newly released DVDs without waiting for markdown prices or discounts [9–11]. He thinks that strategic customers who buy at a discount price and get the most utility through various channels are the devils and believes that strategic customers have brought great losses to the company’s economic benefits.

In terms of the impact of strategic customers on retailers, Liu and Van Ryzin [12] studied the two-period pricing problem of traditional retailers under a single channel. Retailers encouraged customers to buy at full price by reducing inventory at the beginning of sales instead of waiting until the discount period. Compared with the price adjustment of offline B&M stores, online price adjustments are more precise, and the cost of price adjustments is lower than that of offline B&M stores, so we want to know whether these conclusions holds under omni-channel strategies in the presence of rational customers. And if the newsvendor model is extended into supply chain, how do supply chain members’ optimal decisions and profits change?

In this paper, we considered an omni-channel retailer that operates online and B&M stores, online channel, offline channel, and BOPS channel. This paper focuses on a monopoly market and does not consider competition between retailers. The customer is classified into myopic B&M customers and strategic omni-channel customers. The retailer clears the remaining inventory of the B&M store at the end of a sales period through the BOPS channel at a price lower than the online cost. Although on the surface, the markdown strategy will reduce the retailer’s revenue, the research results show that the markdown strategy can increase the retailer’s profit. However, unlike previous studies, the increase in profits is achieved by increasing the inventory of B&M stores. High inventory will stimulate customers to wait patiently for the salvage market because customers can buy online stores. At the same time, with the help of omni-channel information communication, we consider informing customers of the inventory information of B&M stores in advance and promise not to increase inventory during the entire sales period. Markdown strategy will further increase the retailer’s profit based on the price markdown strategy. In addition, we extended the model to the omnichannel supply chain setting. We found that revenue-sharing contracts can coordinate the supply chain but cannot increase the total profit of the decentralized system. The wholesale price contract can increase the total profit of the decentralized system to the optimal profit under the inventory commitment. At the same time, it can coordinate the supply chain through a simple transfer payment.

Our research contributes to the literature in two ways. On one hand, according to the operating characteristics of omni-channel, we established a newsvendor model while considering the markdown price to study how the optimal inventory and the markdown price affect profit, which is rarely used in the omni-channel strategy. Bell et al. [1], Gao and Su [13] considered this factor in their research, but they did not study the issue of a markdown policy. On the other hand, we build the inventory commitment mechanism, which can further improve the retailer’s profit based on a markdown policy model. Also we extended the newsvendor model to supply chain, and find that the wholesale price contract can also increase the profits of the decentralized supply chain, which has the same effect as the inventory commitment mechanism. In Section 2, we review the relevant literature. In Section 3, we establish a fixed price strategy model as the benchmark. Furthermore, we introduce our modeling framework of the markdown strategy and compare it with the benchmark. In Sections 4, we extend the model to the context of the supply chain. In Section 5, we analyze the robustness of our model through numerical research. The 6th section summarizes the full text and proposes future research directions.

## 2 Literature review

This article mainly reviews the literature from two streams of research: Omni-channel strategy; and Markdown pricing with strategic customers.

Brynjolfsson et al. [2] put forward the concept of omni-channel relatively early. He pointed out that in an omni-channel environment, retailers need to adopt new strategies in terms of pricing, resetting customer shopping experience, and establishing better customer relationships to succeed. As an essential BOPS strategy in the omni-channel, many scholars have deeply studied it. Cao et al. [14] studied the impact of BOPS on the demand distribution and profitability of multi-channel retailers. They believe that this new channel can help retailers tap new customer groups and generate additional demand, but it may also weaken the existing channels—by reducing demand and increase operating costs, thereby causing losses to retailers. Gao and Su [13] studied the impact of BOPS channels on store operations and found that BOPS channels are not applicable to all products. Whether its impact on retailers is beneficial or harmful depends on product characteristics. Hu et al. [5] studied the effects of inventory pooling and depooling as well as the impact of different omni-channel strategies. Then discovered that whether a retailer introduces an omni-channel strategy such as BOPS depends on two costs: online waiting costs and store visit costs. Most of their research studies the impact of introducing omni-channel strategies from the inventory perspective but only assumes commodity prices as an exogenous variable. Yang et al. [15] discussed the impact of pricing and customer return probability on inventory management in the omni-channel strategy and compared the profits of the BOPS channel with that of the showroom strategy which provides a reference for the retailer’s omni-channel strategy. Their research on pricing mainly focuses on the myopic customer market to make pricing decisions on commodity prices. Nevertheless, it does not consider the existence of strategic customers in the market.

Wei and Zhang [16] reviewed the impact of strategic customer behavior on corporate decision-making strategies and reviewed potential strategies and decisions to offset the adverse effects of strategic customer behavior. They found that strategic customer behavior mainly affects corporate decision-making in three aspects: pricing, inventory, and information. They then discussed their potential mechanisms against the waiting behavior of strategic customers. Gönsch et al. [17] reviewed the literature on markdown pricing in markets with strategic customers. It mainly reviewed the mechanism of dealing with strategic customers from markdown pricing and systematically classifies and summarizes related papers. Bansal and Maglaras [18] studied the markdown pricing problem of monopolistic companies with strategic customers having different valuations and risk preferences. Mersereau and Zhang [19] studied the two-period pricing problem in a market with an uncertain proportion of strategic customers and found that their robust pricing strategy does not need to consider the strategic level of strategic customers. When strategic customers can learn, Aviv and Pazgal [20] found that the benefits of responsive pricing largely depend on the nature of consumer behavior by comparing with the basic strategy of fixed prices. Lee et al. [21] proposed a model to determine the optimal price reduction timing for companies considering strategic customers’ buying behavior and found that a threshold strategy determines the optimal price reduction timing. Liu and Van Ryzin [12] found that from inventory research, deliberately reducing product inventory to create a quantitative supply risk is the best and thay obtained the best quantity of manufacturing quantitative supply risk. Wu et al. [22] considered markdown pricing strategic, but customers did not know the markdown price exactly when they time their purchases. They conducted numerical studies to investigate the impact of consumers’ knoweledge about the reference price as well as various system parameters on the retailer’s optimal strategies and profitability in the presence of strategic consumers.

However, the previous article mainly studied the markdown pricing issues under a single channel and the inventory and pricing issues for strategic customers under a single channel. Research on strategic customer management and markdown pricing under the omni-channel strategy is still relatively scarce. Unlike the previous research, we have focused on Omni-channel retail, where, including integrating online and offline channels, the retailer can make decisions on both channels simultaneously. Considering the actual situation, when facing strategic customers under omni-channel retail, we aim to study the impact of retailers’ strategies on the BOPS price. Few pieces of research were based on dynamic pricing and inventory in interactive omni-channel strategies. Harsha et al. [3] found that the original price optimization system and the omni-channel strategy are no longer applicable. They proposed a kind of interactive influence. Numerical experiments validate the multi-period stochastic dynamic model for markdown pricing in all channels. Gao and Su [23] reviewed the traditional customer purchase behavior model and introduced a new method of modeling consumer purchase behavior in an omni-channel strategy. Our research has similarities and differences with Harsha et al. [3]’s. The same is that we have considered markdown pricing strategies and inventory decisions under an omni-channel strategy. They proposed a novel multi-stage stochastic program for the markdown pricing of a product in an omni-channel network incorporating the new interactions. Apart from this, they also proposed a deterministic and a robust heuristic where, in each period, omni-channel prices and cross-channel fulfillment inventories are jointly optimized via computationally tractable mixed-integer programs. However, they ignored the existence of strategic customers in the omni- channel strategy; this group of customers would influence retailers’ price and inventory decisions in channel management. Therefore, we make pricing and inventory decisions for omni-channel strategies with strategic customers. Although Liu and Van Ryzin [12], Liu and Van Ryzin [24], Su and Zhang [25], Su and Zhang [26] all studied the impact of markdown pricing on supply chain and retail revenue management in the strategic customer market, we consider implementing markdown pricing strategies for online prices in the omni-channel strategy. However, some retailers may set the same price for different channels. In the study of Gao and Su [13], Zhang et al. [27], it also assumed that the prices of different channels are the same but without considering inconsistent pricing strategies between online prices and offline prices. Our article explores the impact of using markdown pricing strategies on online pricing decisions under different pricing strategies and discusses which pricing strategy is most beneficial to retailers. Table 1 summarizes the model assumptions of the main references.

**Table 1:** Classification of main literatures.

Contributions	Channel	Customer classification	Customer property
Hu(2021)	Omni-channel	Yes	Homogeneous
Liu & Van (2008)	Single channel	No	Heterogeneous
Su & Zhang (2008)	Single channel	No	Homogeneous
This paper	Omni-channel	Yes	Homogeneous

At present, there are many research articles on supply chain contract coordination, among which the most representative one can refer to Cachon [28], Cachon and Lariviere [29]. The research primarily focuses on designing different contractual relationships between suppliers and retailers to maximize the profit or efficiency of the entire supply chain. However, in the omnichannel with strategic customers, the research on contract design is worth studying.

## 3 Model

A monopoly omni-channel retailer operates an offline channel, an online channel, and a buy online and pick up in-store(BOPS) channel. There are two different types of customers in the market: B&M store customers and omni-channel customers. Some empirical studies inspire this customer classification, such as De Keyser et al. [30] and Konuş et al. [31]. B&M store customers are those elderly who do not understand online shopping and cannot use smartphones, and young people who like traditional shopping and loyal to shopping. While omni-channel customers are young people who like to try new products, seek new experiences.

The B&M store customer and omni-channel customer arrive at the same time. The overall demand is uncertain, but the realized demand of the two types of consumers are proportional to each other. Assuming that the demand of the B&M store customer is *θD*, and the demand of the omni-channel customer is (1−*θ*)*D*, where *D* is the total customer random demand. Let us denote the distribution and density of *D* by *F* and *f*, respectively. We assume that the demand distribution has an increasing failure rate, that is, *f*(*x*)/(1−*F*(*x*)) is increasing in *x*; this assumption ensures that the profit function is quasi-concave in classical newsvendor problem and is satisfied by many common probability distributions including uniform, weibull, and truncated normal distribution.

Retailers use B&M store inventory to satisfy B&M store customers and omni-channel customers. This paper assumes that B&M store inventory will prioritize B&M store customers and then BOPS customers as much as possible by using the remaining inventory, It is reasonable to give priority to satisfies the demand of customers with higher profit margins, but it is difficult for retailers in reality because the arrival of both types of customers is random, and we do not know which type of customer will arrive first. In fact, B&M store will reserve inventory for the two types of customers in advance. We consider this situation in the expansion and get similar results as under this assumption. This assumption has also been used in many studies, such as Hu et al. [5], Jalilipour Alishah et al. [32] and this assumption helps simplify our model. *c*_*s*_ and *c*_*o*_ represent the retailer’s unit fulfillment costs of the B&M store and online store (including production costs, storage costs, transportation costs, etc), respectively. The price *p*_*s*_ is the price when B&M store customers choose to buy in the B&M store through offline channel; The price *p*_*o*_ is the price of BOPS channel and online channel when there is no markdown (in the stage of no markdown, the price for omni-channel customers who choose online channels and BOPS channels are both *p*_*o*_). Leftovers at the end of selling season can be sold at a markdown price *p*_*c*_ through BOPS channel (Due to B&M customers are myopic, they will definitely choose price *p*_*s*_ to purchase, so there is no doubt that the retailer will not show the leftovers to the B&M store customers, but show it to omni-channel customer through BOPS channel, which is the unique information effect of the BOPS channel, thence retailer clear the leftover (if remaining) in the B&M store at a markdown price *p*_*c*_ to omni-channel customer through BOPS channel). *p*_*s*_ and *p*_*o*_ are given exogenously and remain unchanged throughout the sales period, only the BOPS channel price drops from *p*_*o*_ to markdown price *p*_*c*_ at the end of the sales period. The key point is that omni-channel customers can only check whether there are leftovers in the B&M store through the smart terminal at the markdown period. If there are leftovers in the B&M store, omni-channel customers choose to place an order online with price *p*_*c*_, and choose to go to pick-up in B&M store, if there are no leftovers in the B&M store, the omni-channel customer can only choose to transfer to the online channel, place an order at the price *p*_*o*_, and choose online delivery). Assuming that the retailer will set *p*_*c*_<*c*_*o*_. Customers’ value is always *v*, *h*_*s*_ is the hassle cost when omni-channel customers pick up in-store after buying online (store visit cost), and *h*_*o*_ is the hassle cost when omni-channel customer purchase through online channel (online waiting cost). If *p*_*s*_+*h*_*s*_≤*p*_*o*_+*h*_*o*_, all strategic customers will purchase through offline channel, there is no need for BOPS channel, and our research will be meaningless. So we assume *p*_*s*_+*h*_*s*_>*p*_*o*_+*h*_*o*_.

In this model, the retailer will determine the markdown price *p*_*c*_ and the inventory quantity *q*. Note that customers can only observe the markdown price but can not see the inventory quantity. The omni-channel customers choose their reservation price of *r*_*c*_ which is not observed by the retailer. The sequence of events for retailer and customer is as follows: first, the retailer privately forms belief ${\hat{r}}_{c}$ over customer’s reservation price in the salvage market and then chooses the optimal inventory *q* and markdown price *p*_*c*_, given these beliefs. Then customers form beliefs $\hat{\xi }$ over the probability that they can get the product on the salvage market and then form their reserve price *r*_*c*_ based on these beliefs. Subsequently, the random demand *D* is realized, then leftovers’ sales occur (if remaining) at markdown price *p*_*c*_. Ultimately, the unsatisfied omni-channel customer needs to transfer to online channel to buy at price *p*_*o*_.

**Fig 1:**
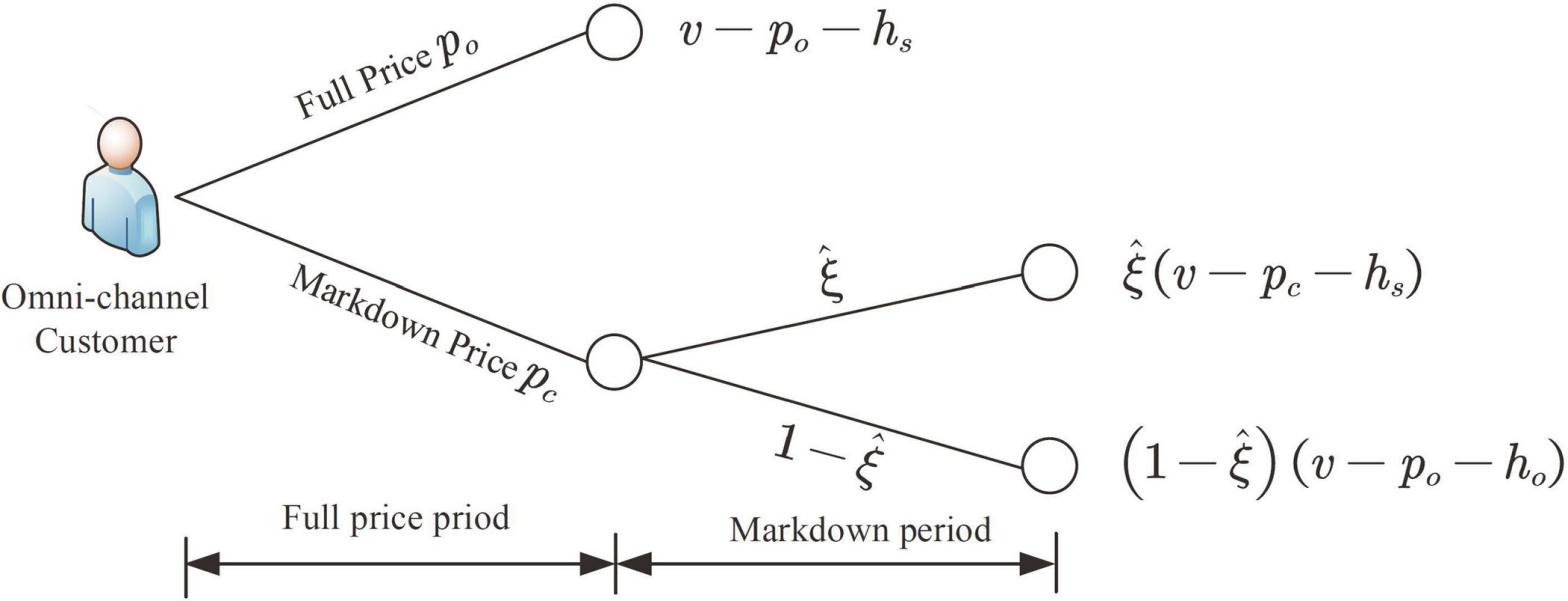
Markdown policy model.

We first describe the customer’s decision problem. **Fig 1** is the omni-channel customer’s decision model.

For B&M store customers, since the price *p*_*s*_ remains unchanged during the entire sales period, all B&M store customers will choose to purchase at *p*_*s*_ to maximize their utility. For omni-channel customers, they will compare the utility obtained at the full price and the markdown price. Consider a omni-channel customer whose expected utility facing the first-period price *p*_*o*_ is *U*_*o*_ = *v*−*p*_*o*_−*h*_*s*_; if he waits to buy, there will be a probability $\hat{\xi }$ to get the product. Based on the belief $\hat{\xi }$, the utility is:

$$U_{o}=\hat{\xi }(v-p_{c}-h_{s})+(1-\hat{\xi })(v-p_{o}-h_{o})$$ (1)

Based on the description above, the surplus value that the customer expects to obtain is:

$$\max\{v-p_{o}-h_{s},\hat{\xi }(v-p_{c}-h_{s})+(1-\hat{\xi })(v-p_{o}-h_{o})\}$$ (2)

Since the omni-channel customer is rational, so only when

$$\hat{\xi }(v-p_{c}-h_{s})+(1-\hat{\xi })(v-p_{o}-h_{o})\geq v-p_{o}-h_{s}\geq 0$$ (3)

Omni-channel customers will choose to buy at a price *p*_*c*_. Given the $\hat{\xi }$, the reservation price of omni-channel customer is $r_{c}(\hat{\xi })$ which is given by the following equation:

$$\hat{\xi }(v-r_{c}(\hat{\xi })-h_{s})+(1-\hat{\xi })(v-p_{o}-h_{o})=v-p_{o}-h_{s}$$ (4)

So we can obtain:

$$r_{c}(\hat{\xi })=p_{o}-h_{s}+h_{o}+\frac{h_{s}-h_{o}}{\hat{\xi }}$$ (5)

Next we consider the retailer’s decision. The two decisions are inventory quantities *q* and the markdown price*p*_*c*_. Given a ${\hat{r}}_{c}$, it’s clear that the retailer determines $p_{c}={\hat{r}}_{c}$ and $q=arg\;max_{q}\Pi_{mp}(q,p_{c})$ where the retailer’s profit function Π_*mp*_ is

$${\Pi_{mp}(q,p_{c})=p}_{s}E[min(\theta D,q)]+p_{o}E[\min\left((1-\theta )D,{\left(q-\theta D\right)}^{+}\right)]1_{\left\{h_{s}\leq h_{o},v-p_{o}\geq h_{o}\right\}}+p_{c}E{\left[{\left(q-\theta D\right)}^{+}-(1-\theta )D\right]}^{+}]-c_{s}q+(p_{o}-c_{o})E[1_{\left\{h_{s}>h_{o}\right\}}(1-\theta )D+1_{\left\{h_{s}\leq h_{o},v-p_{o}\geq h_{o}\right\}}{\left((1-\theta )D-{\left(q-\theta D\right)}^{+}\right)}^{+}]$$ (6)

The subscript “*mp*” in Π_*mp*_(*q*, *p*_*c*_) means “markdown policy”. The first item is the revenue from offline channel purchases by B&M store customers, the second and third items are the revenue from the BOPS channel during the non-price reduction stage and the price reduction stage, and the last item is the revenue from omni-channel customers transferring to online channels when the B&M store stockout at the markdown stage (BOPS channel is not available). Note that omni-channel customers will only choose online channels to buy when *h*_*s*_>*h*_*o*_, thereby leading to two separate parts of online and offline channels. Our model focuses on the potential conflicts between B&M store customers and omni-channel customers when sharing B&M store inventory, so we pay more attention to the case where *h*_*s*_≤*h*_*o*_, *v*−*p*_*o*_≥*h*_*o*_. And this customer classification will not affect the model’s results. Thus, the objective function in (6) can be rewritten as

$${\Pi_{mp}(q,p_{c})=p}_{s}E[min(\theta D,q)]+p_{o}E[\min\left((1-\theta )D,{\left(q-\theta D\right)}^{+}\right)]+p_{c}E{\left[{\left(q-\theta D\right)}^{+}-(1-\theta )D\right]}^{+}]-c_{s}q+(p_{o}-c_{o})E[{\left((1-\theta )D-{\left(q-\theta D\right)}^{+}\right)}^{+}]$$ (7)

To study the strategic interaction between the retailer and the omni-channel customer, we shall use the notion of rational expectation (RE) equilibrium (see, e.g., Cachon and Swinney [33], Su and Zhang [25]). A critical feature of a RE equilibrium is that beliefs must be consistent with actual outcomes. In other words, the retailer’s belief ${\hat{r}}_{c}$ must coincide with customers’ reservation price *r*_*c*_, and customers’ beliefs $\hat{\xi }$ must agree with the actual in-stock probability corresponding to the retailer’s quantity *q*.

**Definition 3.1.** A RE equilibrium (*p*_*c*_, *q*, *r*_*c*_, $\hat{\xi }$, ${\hat{r}}_{c}$) satisfies the following conditions: (Ⅰ) $r_{c}(\hat{\xi })=p_{o}-h_{s}+h_{o}+\frac{h_{s}-h_{o}}{\hat{\xi }}$, (Ⅱ) $p_{c}={\hat{r}}_{c}$, (Ⅲ) $q=arg\;max_{q}\Pi_{mp}(q,p_{c})$, (Ⅳ) $\hat{\xi }=F(q)$, (Ⅴ) ${\hat{r}}_{c}=r_{c}$

Condition (Ⅳ) is given by: $\hat{\xi }=P({\left(q-\theta D\right)}^{+}>(1-\theta )D)=F(q)$. Conditions (Ⅰ), (Ⅱ), and (Ⅲ) assert that under expectation $\hat{\xi }$ and ${\hat{r}}_{c}$, the retailer and omni-channel customers will rationally take actions appropriately in order to reach the maximal utility as discussed above. The final two conditions require that expectations must be consistent with outcomes. In (Ⅳ), the expectation $\hat{\xi }$ must concur with the actual probability of obtaining the product if an individual BOPS consumer waits for the salvage market. This actual probability can be calculated as follows. In equilibrium, the retailer prices the product at BOPS consumers’ reservation price, so all consumers will buy the product. Therefore, if an individual consumer waits instead, this consumer will obtain the product if and only if (*q*−*θD*)^+^>(1−*θ*)*D*, which occurs with probability *F*(*q*), as shown in (Ⅳ). Finally, in (Ⅴ), the retailer must correctly anticipate consumers’ reservation price.

**Proposition 3.1**. In the RE equilibrium, Π_*mp*_(*q*,*p*_*c*_) is concave in *q*, and Π_*mp*_(*q*,*p*_*c*_) has a unique maximizer ($q_{mp}^{*}$, $p_{c}^{*}$) satisfies:

$$\left\{ \begin{array}{c} p_{s}-(p_{s}-c_{o})F(\frac{q_{mp}^{*}}{\theta })+(p_{c}^{*}-c_{o})F(q_{mp}^{*})-c_{s}=0\\ p_{c}^{*}=p_{o}-h_{s}+h_{o}+\frac{h_{s}-h_{o}}{F(q_{mp}^{*})} \end{array}\right.$$ (8)

### 3.1 The basic newsvendor model: No strategic customers

It is instructive to compare the equilibrium inventory quantities and expected profit in our model with that in the classical newsvendor model, where customers are myopic and are willing to pay *v*−*h*_*s*_ for the product under BOPS channel (so the retailer will charges *p*_*o*_ = *v*−*h*_*s*_).

In a market without any strategic customer behavior, the retailer always sets the price *p*_*o*_ = *v*−*h*_*s*_ to achieve the optimal profit under the premise of customer purchase. The retailer’s profit function is:

$${\Pi (q)=p}_{s}E[min(\theta D,q)]+p_{o}E[\min\left((1-\theta )D,{\left(q-\theta D\right)}^{+}\right)]-c_{s}q+(p_{o}-c_{o})E[{\left((1-\theta )D-{\left(q-\theta D\right)}^{+}\right)}^{+}]$$ (9)

**Proposition 3.2**. Π(*q*) is concave in *q* and the uniquely optimal q* satisfies:

$$p_{s}-(p_{s}-c_{o})F(\frac{q^{*}}{\theta })+c_{o}F(q^{*})-c_{s}=0$$ (10)

Our primary research in this paper is the impact of markdown policy with BOPS channel under the omni-channel strategy. It is significant to compare the optimal inventory and optimal pricing before and after implementing the markdown policy.

**Proposition 3.3**. $q^{*}<q_{mp}^{*}$, *and* $\Pi^{*}<\Pi_{mp}^{*}$

According to the studies of Liu and Van Ryzin [20] and Su and Zhang [24], “markdown pricing model” improves the retailer’s profits since the retailer has already restricted availability by stocking at a lower inventory level. This action can encourage customers to buy at full price in an early period. What is different from them is that when confronted with strategic customers, the retailer increases his inventory quantity. And there will be more customers to buy product at markdown period, thereby leading to bigger probability that they can get nothing in markdown period. So, they have to transfer to online channel to purchase (because online inventory is unlimited) to obtain positive customer utility.

### 3.2 Inventory commitment

Can the retailer achieve better results than rationally expected (RE) equilibrium as described above? Su and Zhang [24] proved that inventory and price commitment could help the retailer do better. Although Liu and Van Ryzin [20] did not study from the aspect of inventory commitment, they got the same result as Su and Zhang [24], that is, inventory commitment can increase retailer’s profit.

Here inventory commitment means that the retailer is able to make binding inventory commitment. Inventory commitment may increase the retailer’s profit by guaranteeing to customers that it is not available in large number of quantities, and it cannot be obtained at low prices. Due to the particularity of omni-channel, retailers can convey both price information, and quantity information before omni-channel customers purchase. It is assumed that the retailer can use the commitment mechanism online to help the omni-channel customers, who purchase through the smart terminal, make sure that there are *q* units of inventory in the entire relevant time frame. Omni-channel customers believe that if they wait until the markdown period, they will have a probability *F*(*q*) to get the goods according to the exact observation of the quantity, rather than the probability of stocking based on the rational expectation of the markdown period. So at this time, the reservation price that omni-channel customers are willing to pay is $p_{c}(q)=p_{o}-h_{s}+h_{o}+\frac{h_{s}-h_{o}}{F(q)}$, thence we get the retailer’s profit function for a given committed inventory *q* under the inventory commitment mechanism, which is expressed as:

$${\Pi_{ic}(q)=p}_{s}E[min(\theta D,q)]+p_{o}E[\min\left((1-\theta )D,{\left(q-\theta D\right)}^{+}\right)]+[p_{o}-h_{s}+h_{o}+\frac{h_{s}-h_{o}}{F(q)}]E{\left[{\left(q-\theta D\right)}^{+}-(1-\theta )D\right]}^{+}]-c_{s}q+(p_{o}-c_{o})E{\left[(1-\theta )D-{\left(q-\theta D\right)}^{+}\right]}^{+}=p_{s}q-(p_{s}-c_{o})\theta \int_{0}^{\frac{q}{\theta }}F(x)dx+\left[(p_{o}-h_{s}+h_{o}+\frac{h_{s}-h_{o}}{F(q)})-c_{o}\right]\int_{0}^{q}F(x)dx-c_{s}q+(p_{o}-c_{o})(1-\theta )ED$$ (11)

The subscript “*ic*” stands for “inventory commitment.” Here, under the inventory commitment mechanism, the retailer only needs to make decisions on inventory *q*, this mechanism allows the retailer to actively manipulate the second price *p*_*c*_ as a function of the chosen inventory *q*.

**Lemma 3.1.** When *D* is uniformly distributed, Π_*ic*_(*q*) has a unique maximizer $q_{ic}^{*}$.

The result of Lemma 3.1 holds only when the market demand *D* is uniformly distributed. Unfortunately, it is difficult to obtain the same result for general distributions. Also, we tested two distributions (normal distribution and exponential distribution) in the numerical experiment in the S1 Appendix, the results indicate that the qualitative behavior is also valid.

We next compare the optimal profit of “inventory commitment” with the RE equilibrium profit of “markdown strategy model” with strategic omni-channel customer.

**Proposition 3.4.** $q_{ic}^{*}>q_{mp}^{*}$, and $\Pi_{ic}^{*}>\Pi_{mp}^{*}$

Proposition 3.4 states that inventory commitment increases the retailer’s profits. Also the retailer commits to a stocking quantity $q_{ic}^{*}$ that is higher than the RE equilibrium $q_{mp}^{*}$. Our results are different from those of Su and Zhang [24]. They found that under the classic newsvendor model when the customer is strategic, the retailer’s profit after implementing the inventory commitment mechanism will be higher than that under the RE equilibrium. This is achieved by further restricting inventory quantity. However, our conclusions show that the inventory commitment can also bring higher profits to the retailer under the omni-channel, and it is achieved by setting more inventory than that under the RE equilibrium. This is because in Su and Zhang [24]’s study, when customers face stockout while waiting for the salvage market, they will directly withdraw from the market, and the surplus value obtained is 0, as a result of which, the retailer can use restricting quantity to stimulate strategic customers to buy early at full price period. Whereas, in our model, when a strategic omni-channel customer encounters stockout in the salvage market, they can go to the online channel to purchase directly. They will never encounter stockout, so strategic omni-channel customers will be more “unscrupulous” to wait until the salvage market to buy at a markdown price. Anyway, customers can purchase online when they face stockout. Therefore, the retailer will set a higher inventory to attract strategic customers to purchase in the salvage market. We also analyze these cases numerically in section 6.

## 4 Markdown strategy in supply chains

In the previous section, we got some interesting conclusions from the retailer’s perspective. This section expands the research object from a single retailer to a supply chain that includes one manufacturer and one retailer. Manufacturer distributes their products through retailers to customers. In the supply chain context, whether there is a supply contract that can achieve profits under a decentralized system? The retailer and the manufacturer first sign a purchase contract. Then retailers and customers make decisions on inventory and price based on the equilibrium of rational expectation. (In this section, we also assume that retailers only decide the markdown price *p*_*c*_ and inventory quantity *q*). We will mainly study wholesale price contract and revenue sharing contract.

### 4.1 Revenue sharing contract

We use w to denote the wholesale price signed by the retailer and the manufacturer. Regarding it as an exogenous parameter, which is determined by the market position of manufacturers and retailers. In this paper we will not extend too much. Please refer to Cachon [5] and Cachon and Lariviere [6] for the research on supply chain contracts, both of which show that the revenue sharing contract can coordinate the supply chain.

Before production and sales, the manufacturer signs a contract {*w*, *λ*} with the retailer. *λ*∈(0,1) represents the retailer’s revenue-sharing ratio, and 1−λ represents the manufacturer’s share of the revenue. Then, the retailer’s revenue function and the manufacturer’s revenue function under the revenue sharing contract are given, respectively, by

$$\Pi_{s}^{r}=\lambda \{E[min(\theta D,q)]+p_{o}E[\min\left((1-\theta )D,{\left(q-\theta D\right)}^{+}\right)]+p_{c}E{\left[{\left(q-\theta D\right)}^{+}-(1-\theta )D\right]}^{+}]+(p_{o}-c_{o})E{\left[(1-\theta )D-{\left(q-\theta D\right)}^{+}\right]}^{+}-wq==\lambda \{p_{s}q-(p_{s}-c_{o})\theta \int_{0}^{\frac{q}{\theta }}F(x)dx+(p_{c}-c_{o})\int_{0}^{q}F(x)dx+(p_{o}-c_{o})(1-\theta )ED\}-wq$$ (12)

$$\Pi_{s}^{m}=(1-\lambda )\{p_{s}E[min(\theta D,q)]+p_{o}E[\min\left((1-\theta )D,{\left(q-\theta D\right)}^{+}\right)]+p_{c}E{\left[{\left(q-\theta D\right)}^{+}-(1-\theta )D\right]}^{+}]+(p_{o}-c_{o})E{\left[(1-\theta )D-{\left(q-\theta D\right)}^{+}\right]}^{+}+(w-c_{s})q=(1-\lambda )\{p_{s}q-(p_{s}-c_{o})\theta \int_{0}^{\frac{q}{\theta }}F(x)dx+(p_{c}-c_{o})\int_{0}^{q}F(x)dx+(p_{o}-c_{o})(1-\theta )ED\}+(w-c_{s})q$$ (13)

The superscript “*r*” represents the retailer, the superscript “*m*” represents the manufacturer, and the subscript “*s*” represents the revenue-sharing contract. Revenue-sharing contract can achieve supply chain coordination by making the retailer’s profit function an affine transformation of the supply chains profit function.

**Theorem 4.1. *A set of contracts {w*, *λ}satisfying***

$$w=\lambda c_{s}$$ (14)

***can coordinate the supply chain, the manufacturer’s profit and the retailer’s profit are*** $\Pi_{s}^{r}=\lambda \Pi_{s}(q,p_{c})$, $\Pi_{s}^{m}=(1-\lambda )\Pi_{s}(q,p_{c})$.

Eq (14) indicates that revenue-sharing contracts coordinate the supply chain and arbitrarily allocate profit. The particular profit split chosen probably depends on the firms’ relative bargaining power, which is reflected in the size of *w*. As Cachon and Lariviere [6] said that coordination requires a wholesale price below the supplier’s cost of production *c*_*s*_. The supplier loses money in selling the product and only makes money by participating in the retailer’s revenue. Selling below the cost price is necessary because revenue sharing systematically drops the retailer’s marginal revenue curve below the integrated supply chains. To have marginal revenue equals marginal cost at the desired point, the retailer’s marginal cost must also be less than the integrated system.

### 4.2 Wholesale price contract

A wholesale price contract specifies the unit price *w*(*w*≥*c*_*s*_) that the manufacturer charges the retailer. Here, we consider *w* a proxy of firms’ relative bargaining power and is given exogenously: a higher *w* reflects a more favorable position for the manufacturer. Based on the above description, the profit function of the retailer under the wholesale price contract can be obtained by:

$${\Pi_{w}^{r}(q,p_{c})=p}_{s}E[min(\theta D,q)]+p_{o}E[\min\left((1-\theta )D,{\left(q-\theta D\right)}^{+}\right)]+p_{c}E{\left[{\left(q-\theta D\right)}^{+}-(1-\theta )D\right]}^{+}-wq+(p_{o}-c_{o})E{\left[(1-\theta )D-{\left(q-\theta D\right)}^{+}\right]}^{+}=p_{s}q-(p_{s}-c_{o})\theta \int_{0}^{\frac{q}{\theta }}F(x)dx+(p_{c}-c_{o})\int_{0}^{q}F(x)dx-wq+(p_{o}-c_{o})(1-\theta )ED$$ (15)

In the equilibrium, $\Pi_{w}^{r}(q,p_{c})$ is concave in *q*, *q* has a unique maximizer $\left(q_{w}^{*},p_{w}^{*}\right)$ satisfied:

$$\left\{ \begin{array}{c} p_{s}-(p_{s}-c_{o})F(\frac{q_{w}^{*}}{\theta })+(p_{c}^{*}-c_{o})F(q_{w}^{*})-c_{s}=0\\ p_{c}^{*}=p_{o}-h_{s}+h_{o}+\frac{h_{s}-h_{o}}{F(q_{w}^{*})} \end{array}\right.$$ (16)

Manufacturer profit function is expressed as:

$$\Pi_{w}^{m}(q)=q_{w}^{*}(w-c_{s})$$ (17)

The total profits of the supply chain are $\Pi_{w}=\Pi_{w}^{r}+\Pi_{w}^{m}$, the subscript “*w*” represents the wholesale price contract.

The optimal total profits in the decentralized system is:

$$\Pi_{w}^{*}=\Pi_{w}^{r*}+\Pi_{w}^{m*}==p_{s}q_{w}^{*}-(p_{s}-c_{o})\theta \int_{0}^{\frac{q_{w}^{*}}{\theta }}F(x)dx+(p_{c}^{*}-c_{o})\int_{0}^{q_{w}^{*}}F(x)dx-c_{s}q_{w}^{*}+(p_{o}-c_{o})(1-\theta )ED=p_{s}q_{w}^{*}-(p_{s}-c_{o})\theta \int_{0}^{\frac{q_{w}^{*}}{\theta }}F(x)dx+\left[(p_{o}-h_{s}+h_{o}+\frac{h_{s}-h_{o}}{F(q_{w}^{*})})-c_{o}\right]\int_{0}^{q_{w}^{*}}F(x)dx-c_{s}q_{w}^{*}+(p_{o}-c_{o})(1-\theta )ED$$ (18)

It can be deduced from Eq (16) that the optimal inventory quantity is monotonically decreasing in the wholesale price, We can easily know by implicit theorem that $dp/dw=-1/\left[(p_{s}-c_{o})f(q/\theta )/\theta -(p_{o}-h_{s}+h_{o}-c_{o})f(q)\right]<0$ when demand follows uniform distribution. For other distributions, we verified our qualitative insights with numerical values in the S6 and S7 Figs in S1 Appendix. That is to say, the wholesale price signed by the retailer and the manufacturer under the RE equilibrium can determine the only inventory quantity and the markdown price. Therefore, we can determine that the value range of *w*≥*c*_*s*_. *w*≥*c*_*s*_ ensure that the manufacturer’s profit is nonnegative, that is, the manufacturer is willing to sell the product when the wholesale price *w*≥*c*_*s*_. As mentioned above, there is a one-to-one relationship between $q_{w}^{*}$ and *w*. Therefore, by varying *w*≥*c*_*s*_, the system can realize any equilibrium quantity within the corresponding range. Although this particular quantity has to conform to the requirements of an RE equilibrium. In this sense, a wholesale price contract to the supply chain is similar to the profit growth effect of inventory commitment to retailer.

**Proposition 4.1.** For any given *w*≥*c*_*s*_, the equilibrium total profits in the decentralized system is less than or equal to the markdown model profit, i.e., $\Pi_{w}^{*}\leq \Pi_{mp}^{*}$.

**Fig 2:**
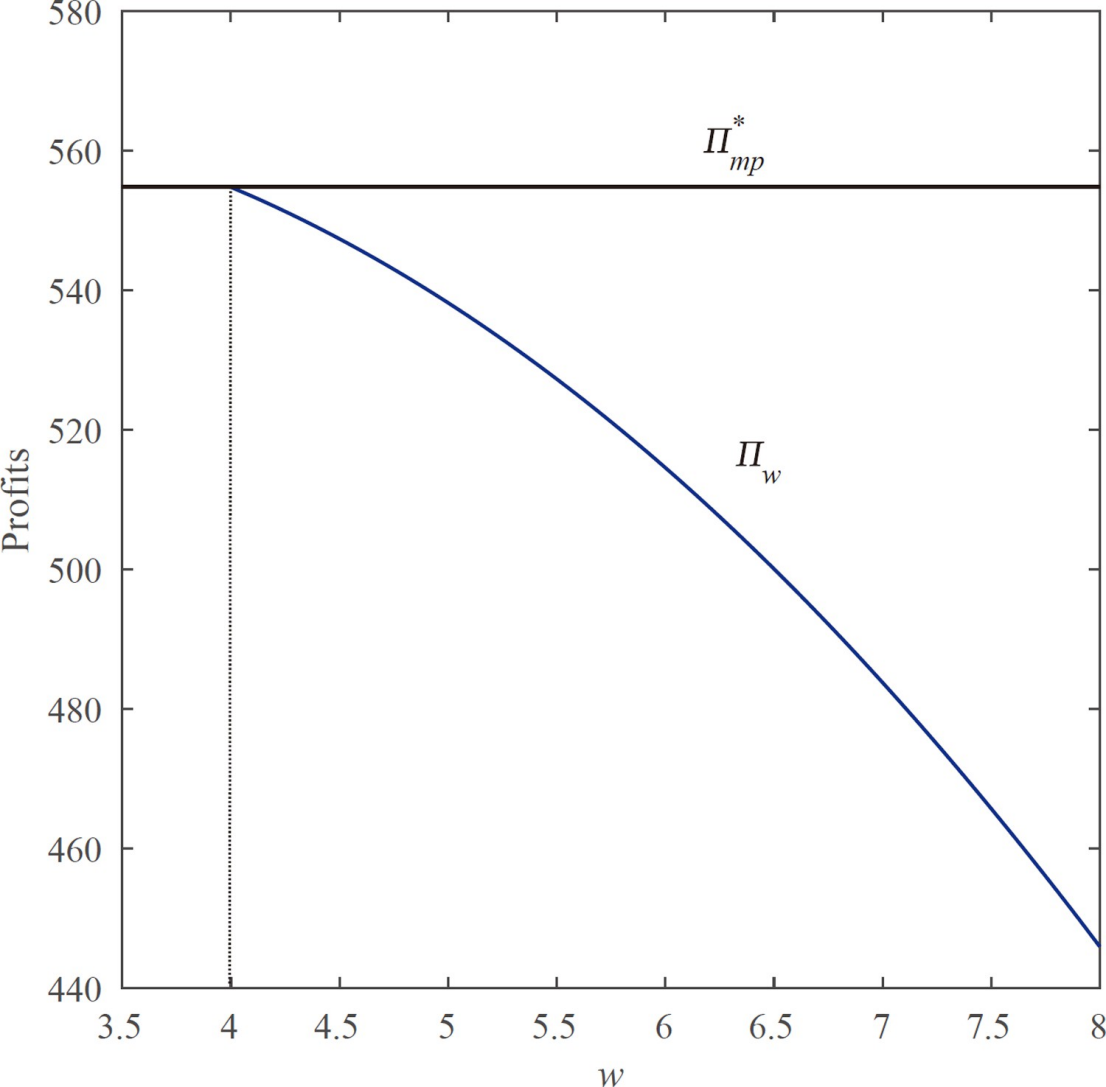
Comparison of the profit function of $\Pi_{mp}^{*}\;and\;\Pi_{w}$. *Demand follows a uniform distribution U (0,100), and parameter values are p*_*s*_ = *15*, *p*_*o*_ = *11*, *c*_*o*_ = *5*, *c*_*s*_ = *4*, *θ* = *0.5*, *h*_*o*_ = *3*, and *h*_*s*_ = *1*.

Proposition 4.1 indicates that the total profit of the decentralized system under the supply chain setting is less than or equal to the optimal profit ($\Pi_{mp}^{*}$). This is because the inventory quantity and price decrease as the wholesale price increases which will reduce the total profit of the decentralized system. As the **Fig 2** shows:

We find that the manufacturer will gain profit and choose to sign the contract with the wholesale price *w* only when *w*≥*c*_*s*_. We want to know whether there is a wholesale price lower than *c*_*s*_ that makes the total profit of the decentralized system in the supply chain higher than the optimal profit under the centralized system and even obtains the optimal profit under the inventory commitment. Thus, we have the following proposition.

**Proposition 4.2.** For any given *w*, there exists uniquely optimal w* of the equilibrium total profit in the decentralized system. Also the equilibrium total profit is greater than the profit under markdown policy, i.e., $\Pi_{w}^{*}>\Pi_{mp}^{*}$.

**Fig 3:**
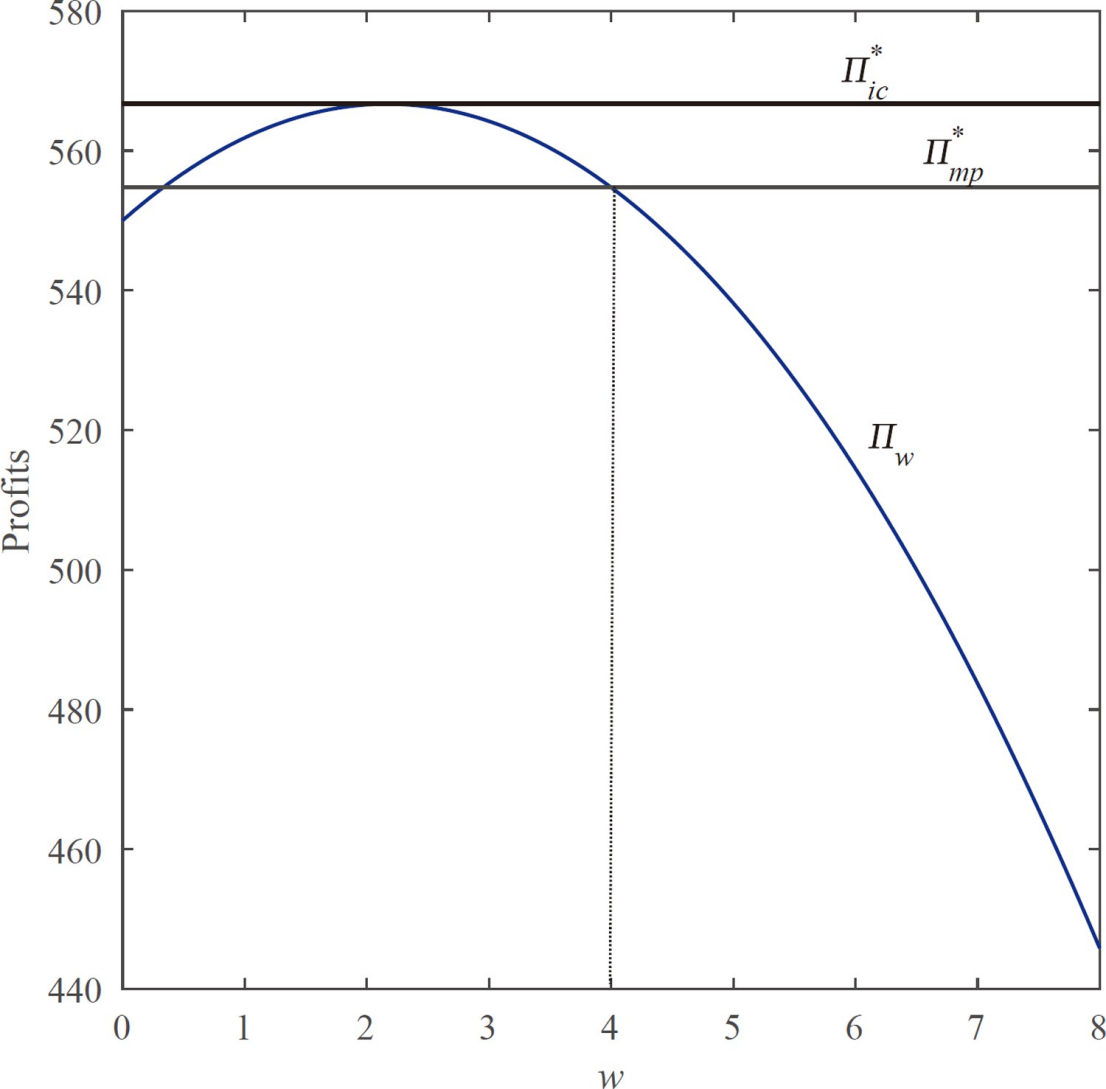
Comparison of the profits function with respect to *w*. *Demand follows a uniform distribution U [0,100], and parameter values are p*_*s*_ = *15*, *p*_*o*_ = *11*, *c*_*o*_ = *5*, *c*_*s*_ = *4*, *θ* = *0.5*, *h*_*o*_ = *3*, *and h*_*s*_ = *1*.

In order to evaluate the robustness of our results, we tested the analysis results with simulation analysis as shown in **Fig 3**. As we all know, the profit of the decentralized supply chain under the wholesale price contract is always lower than the profit under the centralized system due to the double marginal effect. Nevertheless, the proposition gives the opposite conclusion. The total profit of the decentralized supply chain is greater than that under the centralized system by decreasing the wholesale price. This is due to the existence of strategic omni-channel customers. When the retailer decreases the wholesale price, the optimal order quantity and the markdown price under the RE equilibrium will increase, thereby increasing the overall profit of the supply chain. As two independent firms (the retailer and the manufacturer) in the de- centralized system, the wholesale price contract cannot coordinate the supply chain due to the double marginal effect. However, the wholesale price contract can increase the total profits of the decentralized system to the optimal, which is equal to the inventory commitment mechanism. Thence, for two absolute rational, independent firms, they are motivated to achieve the optimal supply chain through external compensation mechanisms; that is, the retailer needs to pay the manufacturer at least $q^{*}(c_{s}-w^{*})$ to make the manufacturer’s profit positive. Only in this way, will the manufacturer accept a wholesale price lower than the production cost; this is also profitable for retailer. This coordination is only achieved through transfer payment which is different from the revenue sharing contract.

## 5 Numerical studies

We will discuss the influence of exogenous variables on the retailer’s optimal inventory, optimal markdown price, and optimal profit. In this paper we focuse on *p*_*s*_, *p*_*o*_, *c*_*o*_, and *p*_*s*_−*c*_*s*_ indicates the profit margin of the B&M store as well as reflects the retailer’s B&M store operating capabilities. *p*_*o*_−*c*_*o*_ is the profit margin of online channel, and it also reflects the retailer’s online channel operating capabilities. po is the full price of the online channel. We use an numerical analysis to illustrate Proposition 3.1. Let the market demand follows an uniform distribution *D* obeys U [0, 100], *p*_*s*_ = 15, *h*_*o*_ = 3, *h*_*s*_ = 1. Other parameters are spanned by:

$$\theta \in \{0.3,0.5\},c_{o}\in \{5,6,7\}$$

$$c_{s}\in \{3.5,4,4.5,5,5.5,6,6.5,7,7.5\}$$

$$p_{o}\in \{10,11.5,12,12.5,13,13.5,14,14.5\}$$

**Fig 4:**
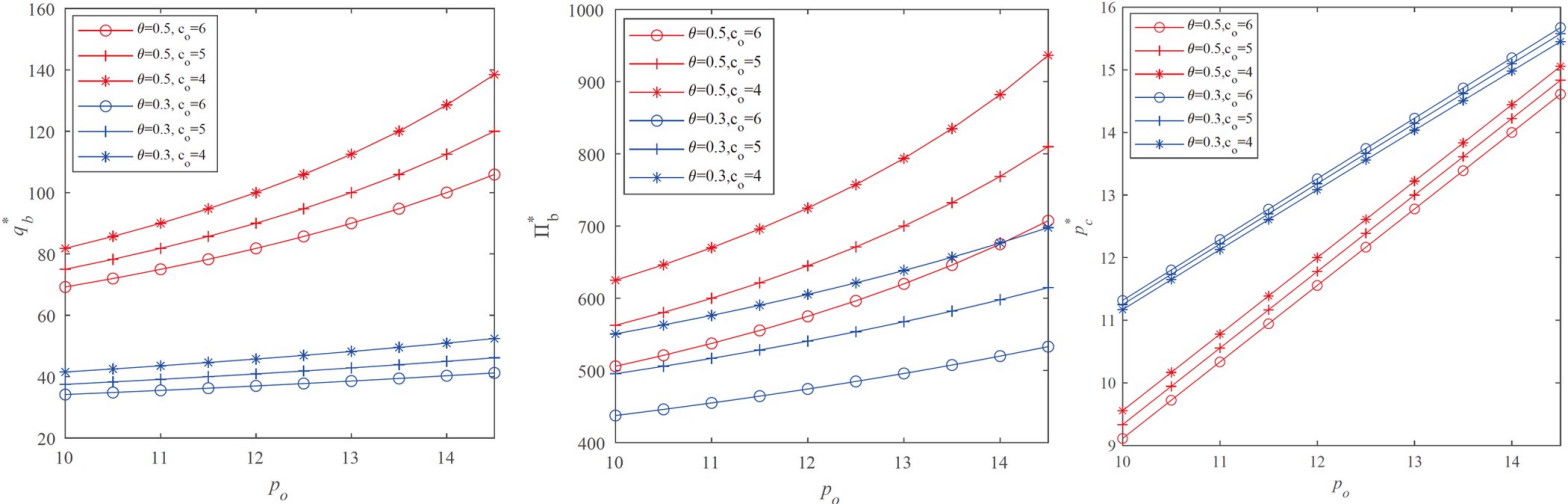
Impact of full price on the retailer’s optimal decision.

The pattern of the firm’s optimal stocking quantity $q_{mp}^{*}$ and retailer’s profit $\Pi_{b}^{*}$ with increasing the B&M store product price *p*_*o*_ are shown in **Fig 4** with the different unit fulfillment costs of the online store and the percentage of B&M store customers.

We observe that the optimal stocking quantity $q_{mp}^{*}$ and expected profit $\Pi_{mp}^{*}$ are increasing in *p*_*o*_. When the first online full price is relatively high, the retailer needs to store more inventory to obtain higher revenue. This result is reasonable because on the one hand, the higher po and higher availability of inventory $F(q_{mp}^{*})$ prompts strategic customers to wait to buy at a lower price when they face stockout in the salvage market, and they switch to online channels to buy. At this time, the retailer gets *p*_*o*_−*c*_*o*_ income per product. On the other hand, the optimal stocking quantity $q_{mp}^{*}$ and expected profit $\Pi_{mp}^{*}$ are increasing in *θ*. In other words, when the number of strategic customers in the market decreases, the retailer’s profits increase, which is consistent with reality.

**Fig 5:**
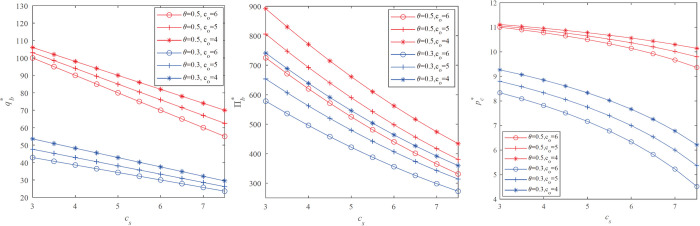
Impact of the unit fullfillment costs of the B&M store *c*_*s*_ on the retailer’s optimal decision.

We observe from **Fig 5** that the optimal stocking quantity $\Pi_{mp}^{*}$ and the optimal profit $\Pi_{mp}^{*}$ are decreasing in *c*_*s*_. In other words, when other parameters are fixed, the retailer’s optimal inventory and optimal profit will decrease with the unit fulfillment cost of the B&M store. This indicates that when the retailer’s B&M store cost is higher, the retailer will keep the offline price unchanged and store less and less inventory. We did not analyze the profit margin of B&M store *p*_*s*_−*c*_*s*_ and the B&M store price *p*_*s*_. This is because *p*_*s*_−*c*_*s*_ and *p*_*s*_ have exactly the opposite properties with cs, so we will not repeat them.

The optimal inventory and expected profit under the three models of the markdown strategy model, single price model, and inventory commitment strategy are calculated for each parameter combination. Fig 5 shows the profit changes in the different proportion of strategic customers and the different costs *c*_*o*_, *c*_*s*_. First, we observed that the profit of the inventory commitment model is always greater than the optimal profit under the markdown strategy model, and the optimal profit under the markdown strategy mode is always greater than the optimal profit under the fixed price strategy model. Therefore, in an omni-channel environment, when there are more strategic customers in the market, the inventory commitment strategy will bring more profits to the retailer. This is intuitive because under the omni-channel strategy, implementing a limited supply of B&M store inventory will aggravate the wait-for-purchase behavior of strategic customers. Next, we observed that in the fixed price strategy model, markdown strategy model, and inventory commitment model, the retailer’s profit decreases monotonically with the unit fulfillment costs of the B&M store *c*_*s*_. It is natural that the higher the production cost is, the lower the profit will be. But when there are fewer strategic customers, we found that the total profit of the markdown strategy model and the inventory commitment model increases as the fulfillment cost of the online store co increases. This is because higher online fulfillment costs will encourage retailers to set up more inventory in B&M stores, which improves the retailers’ profits to a certain extent.

## 6 Extensions

In the previous model analysis, we assumed that the B&M store inventory would give priority to fulfill the B&M store customers, and the remaining inventory would then fulfill the BOPS channel demand. To make the model reasonable, we will consider that B&M store customers and omni-channel customers have equal inventory service priorities. In this section, we use proportional distribution rules to show that the two types of customers have equal service priorities. We also pay more attention to the case with *h*_*s*_≤*h*_*o*_, *v*−*p*_*o*_≥*h*_*o*_. In this case, omni-channel customers will first buy through the BOPS channel. With the equal priority, the retailer’s profit function without strategic customers can be expressed as

$${\bar{\Pi }(q)=p}_{s}E[min(\theta D,\theta q)]+p_{o}E[\min\left((1-\theta )D,(1-\theta )q\right)]-c_{s}q+p_{s}E[min\;\theta {\left(D-q\right)}^{+},(1-\theta ){\left(q-D\right)}^{+}]+p_{o}E[min\;\theta {\left(q-D\right)}^{+},(1-\theta ){\left(D-q\right)}^{+}]+(p_{o}-c_{o})E{\left[(1-\theta ){\left(D-q\right)}^{+}-\theta {\left(q-D\right)}^{+}\right]}^{+}$$ (19)

Similarly, we can also get the retailer’s profit function under the markdown policy with strategic customers

$${{\bar{\Pi }}_{mp}(q,p_{c})=p}_{s}E[min(\theta D,\theta q)]+p_{o}E[\min\left((1-\theta )D,(1-\theta )q\right)]-c_{s}q+p_{s}E[min\;\theta {\left(D-q\right)}^{+},(1-\theta ){\left(q-D\right)}^{+}]+p_{o}E[min\;\theta {\left(q-D\right)}^{+},(1-\theta ){\left(D-q\right)}^{+}]+p_{c}E{\left[\theta {\left(D-q\right)}^{+}-(1-\theta ){\left(q-D\right)}^{+}\right]}^{+}+p_{c}E{\left[\theta {\left(q-D\right)}^{+}-(1-\theta ){\left(D-q\right)}^{+}\right]}^{+}+(p_{o}-c_{o})E{\left[(1-\theta ){\left(D-q\right)}^{+}-\theta {\left(q-D\right)}^{+}\right]}^{+}$$ (20)

We use $\bar{\Pi }(q)$, ${\bar{\Pi }}_{mp}(q)$ respectively to represent the optimal profits of the fixed price model and markdown model with the equal priority, and ${\bar{q}}^{*}$, ${\bar{q}}_{mp}^{*}$ represent the optimal inventory.

We also consider the inventory commitment strategy under the equal fulfill priority. The reservation markdown price that omni-channel customers are willing to pay is $p_{c}=p_{o}-h_{s}+h_{o}+\frac{h_{s}-h_{o}}{F(q)}$, thence we get the retailer’s profit function for a given commitment inventory *q* under the inventory commitment mechanism, which is expressed as:

$${{\bar{\Pi }}_{ic}(q)=p}_{s}E[min(\theta D,\theta q)]+p_{o}E[\min\left((1-\theta )D,(1-\theta )q\right)]-c_{s}q+p_{s}E[min\theta {\left(D-q\right)}^{+},(1-\theta ){\left(q-D\right)}^{+}]+p_{o}E[min\theta {\left(q-D\right)}^{+},(1-\theta ){\left(D-q\right)}^{+}]+[p_{o}-h_{s}+h_{o}+\frac{h_{s}-h_{o}}{F(q)}]E{\left[(1-\theta ){\left(q-D\right)}^{+}-\theta {\left(D-q\right)}^{+}\right]}^{+}+[p_{o}-h_{s}+h_{o}+\frac{h_{s}-h_{o}}{F(q)}]E{\left[\theta {\left(q-D\right)}^{+}-(1-\theta ){\left(D-q\right)}^{+}\right]}^{+}+(p_{o}-c_{o})E{\left[(1-\theta ){\left(D-q\right)}^{+}-\theta {\left(q-D\right)}^{+}\right]}^{+}$$ (21)

We use ${\bar{\Pi }}_{ic}(q_{ic})$ to represent the optimal profits of the inventory commitment model with the equal priority, and ${\bar{q}}_{ic}$ represent the optimal inventory.

**Proposition 6.1.** ${\bar{q}}^{*}<{\bar{q}}_{mp}^{*}<{\bar{q}}_{ic}^{*}$, and ${\bar{\Pi }}^{*}<{\bar{\Pi }}_{mp}^{*}<{\bar{\Pi }}_{ic}^{*}$

Comparing Proposition 3.3 and Proposition 6.1, it can be found that under the equal fulfill priority, the markdown strategy can still obtain higher profits by increasing the inventory of B&M stores. Comparing Proposition 3.3 and Proposition 6.1, the inventory commitment strategy can still further increase the profit of the retailer under the equal fulfill priority. Combined with Proposition 6.1, equal fulfill priority will not change our qualitative insights in this paper. In other words, when the fulfill priority of omni-channel customers increases and the service priority of B&M store customers decreases, our main results remain valid. This is because the omni-channel customers demand are fulfilled by proportionally allocated inventory under the same fulfill priority. When retailers increase B&M store inventory, the corresponding fulfill omni-channel customers’ inventory also increases, so more omni-channel customers are fulfilled. Omni-channel customers are willing to wait until the price is cleared to buy. And after the corresponding B&M store inventory is processed, the B&M store customers will switch to online channels where the retailer is more profitable and will get higher profits. This improvement has the same mechanism of prioritizing B&M stores customers.

## 7 Conclusion and remark

The waiting behavior of strategic customers has a significant influence on the retailer’s decision. Retailers should consider this behavior when pricing and making inventory decisions to effectively reduce the loss caused by the waiting for behavior. In particular, we studied the pricing and inventory decision-making issues under the retailer’s omni-channel strategy. Our model assumes that customers have the same value as the retailer’s products, but the arrival of customers is random. Customers are divided into myopic B&M stores customers and strategic omni-channel customers. The research results show that the markdown strategy can improve retailers’ profits. However, our results are different from Liu and Van Ryzin [12]; Su and Zhang [25]. In Liu and Van Ryzin [12], They found strategic customers can be motivated to buy at full price in advance, thereby increasing retail profits by creating inventory rationing risk. However, this paper found that in the context of omni-channel, increasing inventory to attract strategic customers to delay purchases can improve retailers’ profits. In Su and Zhang [25], they found that under the classic newsvendor model when the customer is strategic, the retailer’s profit after implementing the inventory commitment mechanism will be higher than that under the RE equilibrium. This is achieved by further restricting inventory quantity. However, our conclusions show that the inventory commitment can also bring higher profits to the retailer under the omni-channel, and it is achieved by setting more inventory than that under the RE equilibrium. High inventory will stimulate customers to wait more patiently for the salvage market because customers can turn to online channels to buy (because the inventory of online store is unlimited). In the previous single-channel model, customers can only withdraw from the market and obtain zero surplus value when B&M store is stockout. Therefore, low inventory rationing stimulates customers to purchase at full price. It is precise because of the markdown strategy that attracts more strategic customers into the second phase. Therefore, when the online operations of omnichannel retailers are relatively good, retailers can adopt the online markdown strategy. However, if the retailer’s online operations are poor, this strategy will hurt the retailer’s profits. At the same time, it is found that retailers under the omni-channel background can inform customers of the inventory quantity before the sales period and promise in some way (retailer’s reputation or brand effect) that the inventory will not increase in the future sales period. This will further increase the profits of retailers. Therefore, the inventory commitment mechanism is not only beneficial to retailers under traditional channels, but can also enable retailers to obtain greater profits under omni-channel, the inventory commitment mechanism.

We extended the model to the supply chain setting and also got some significant conclusions. The total profit of the decentralized system under the supply chain setting can reach the optimal profit under the centralized system, and a revenue-sharing contract can achieve coordination between the manufacturer and the retailer so far. Although traditional research shows that wholesale price contracts cannot coordinate the supply chain, we have found that in an omni-channel supply chain with strategic customers, wholesale price contracts can increase the total profit of the decentralized system to the optimal profits under inventory commitment. It is similar to the power of inventory commitment, and the supply chain can be coordinated through a simple transfer payment.

Some work needs to be done in future research. First, we can continue our work and relax some assumptions. Secondly, this article focuses on the situation of monopolistic retailers and two types of customers. Another situation that should be considered is the competition among multiple retailers as well as the competition for customer market. In addition, considering the uncertainty of supply chain and manufacturer’s production is also an exciting direction in the future.

## Supporting information

S1 Appendix (DOCX)
