# Supplementary material for: Omni-channel retail operations in the presence of strategic customers: The benefit of inventory commitment
Source: PLoS One. 2022 May 5;17(5):e0264900. doi: 10.1371/journal.pone.0264900 (PMC9070910; doi:10.1371/journal.pone.0264900)
Supplement: S1 Appendix — (DOCX) [file pone.0264900.s001.docx]

**Appendix**

**Proof of Proposition 3.1**

The retailer’s profit function under the markdown strategy model is:

So we can obtain

and

Under

there exsits the optimal make , that is . In the RE equilibrium,

So we can obtain the result:

|  | (22) |
| --- | --- |

**Proof of Proposition 3.2**

When , the retailer’s expected profit under the fixed price strategy model is:

So we can obtain

and

We know that , there exsits the optimal make , it means that .

In the RE equilibrium, . So we can obtain the result:

When , the retailer’s expected profit is:

The proof is similar to the case when , the result is proven

|  | (23) |
| --- | --- |

**Proof of Proposition 3.3**

From the above, the first derivative of the profit function of the fixed price strategy with respect to is

The first derivative of the profit function of the markdown strategy with respect to is

Because:

When inventory is zero, . So we only need to compare the value of the first derivative of the two profit functions.

The derivative of at is:

is satisfied since the two profit functions are concave functions. Proposition 3.3 is proved.

**Proof of Lemma 3.1**

When D is uniformly distributed with [0,A], the derivative of with respect to can be written as

and taking derivative again with respect to gives:

The last inequality can be obtained by . We know that , is concave in , has a unique maximizer . Lemma 3.1 has been proved.

**Proof of Proposition 3.4**

From Eq. (16), the first derivative of the profit function under the inventory commitment is:

Compare with

So we can obtain: .

at is

|  | (24) |
| --- | --- |

From the previous lemma we know that Π*_ic_* and Π*_b_* are both increasing first and then decreasing in . Hence , .

**Proof of Proposition 4.1**

The retailer’s profit in the decentralized system, under rational expectation equilibrium which is as follows:

|  | (25) |
| --- | --- |

while the manufacturer’s optimal profit function is:

|  | (26) |
| --- | --- |

The total profits in the decentralized system under the wholesale price contract is:

|  | (27) |
| --- | --- |

The first derivative of the retailer’s profit function is:

|  | (28) |
| --- | --- |

Make the first derivative of the retailer’s profit function equal to 0 (the retailer’s profit

function is cancave), reduce to:

|  | (29) |
| --- | --- |

where .

Substituting Eq. (29) into the retailer’s profit function Eq. (25) and manufacturer’s profit function Eq. (26), we get:

and .

The optimal total profits in the decentralized system is:

|  | (30) |
| --- | --- |

The retailer’s profit function for a given commitment inventory q under the inventory commitment mechanism is expressed as:

|  | (30) |
| --- | --- |

Compared with , it’s shows that there exsits the correspondingly unique enables the total profits in the decentralized system to achive the optimal profit under inventory commitment for the optimal . Next we consider whether the above conclusion is true or not when . When , we can get from Eq. (24)

We can know from Eq. (29) that decreases as w increases. That is to say can only achieve the maximum value which euqal to since ww has a limited range, cannot achieve the optimal profit under the inventory commitment mechanism. Combine with proposition 3.4, the results are proven.

**Proof of Proposition 6.1**

The first derivative of the profit function of the fixed price strategy with respect to is

The first derivative of the profit function of the markdown strategy with respect to is:

The first derivative of the profit function under the inventory commitment is

Because:

So we can obtain:.

When inventory is zero, . So we only need to compare the value of the first derivative of the two profit functions. The derivative of at is:

the first derivative of of the profit function under the inventory commitment at is:

is proven.

**Fig 6: Impact of the Wholesale on the Retailer’s Optimal Inventor**


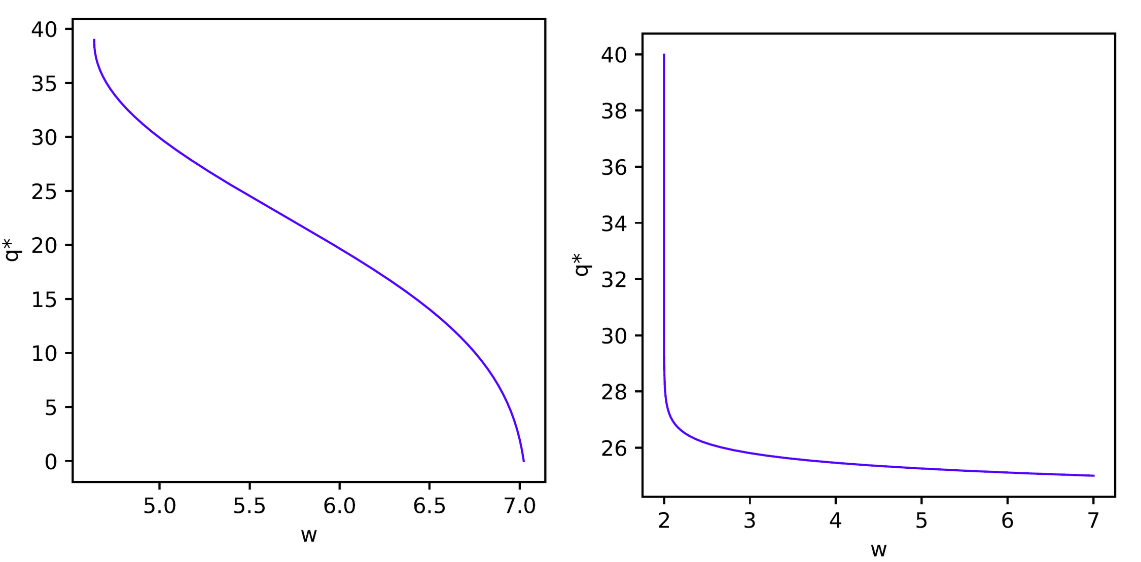
A. When *D* Obbey Normal Distribution *N*(50*,* 25)

B. When *D* Obbey Exponential Distribution *E*(0*.*02)

***Note. Parameter values are =10, =8, =5, =0.5, =4, and =1.***

**Fig 7: Impact of the Wholesale on the Retailer’s Optimal Profit**


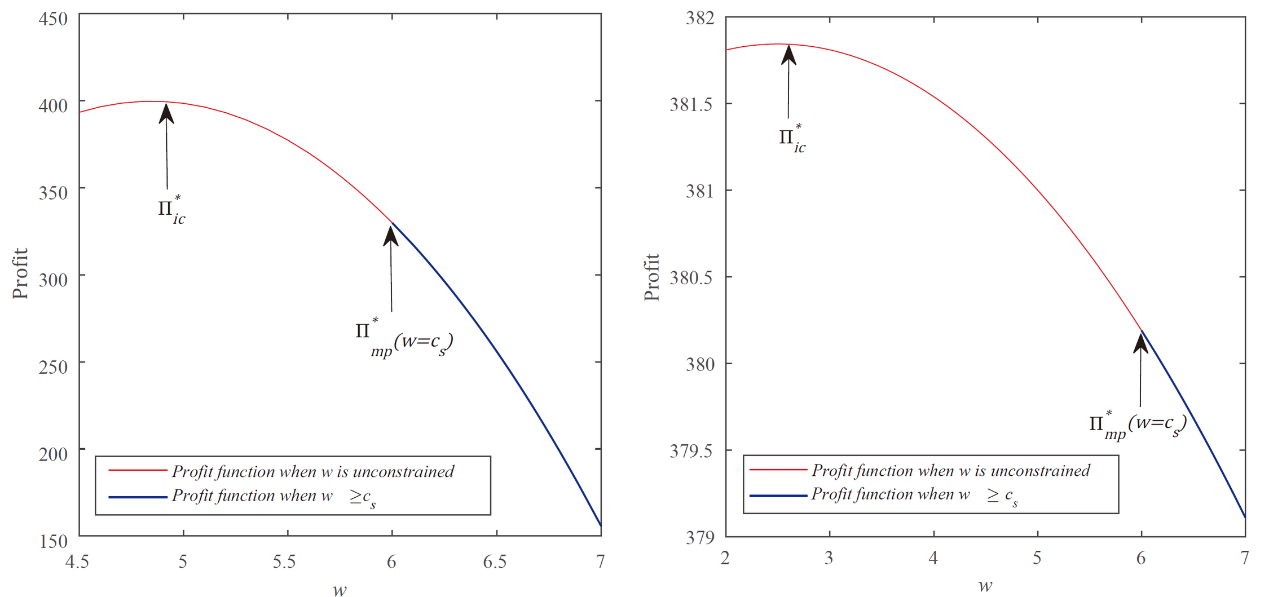


A. When *D* Obbey Normal Distribution *N*(50*,* 25)

B. When *D* Obbey Exponential Distribution *E*(0*.*02)

***Note. Parameter values are =10, =8, =5, =0.5, =4, and =1.***
